# A Fatal Case Involving Chronic Intravenous Use of Homemade Methcathinone Derived from Pseudoephedrine Tablets: Post-Mortem Concentrations, Health Risk, and Medicolegal Aspect

**DOI:** 10.3390/ijms262411974

**Published:** 2025-12-12

**Authors:** Karolina Nowak, Paweł Szpot, Marcin Zawadzki

**Affiliations:** 1 Department of Pharmacology, Faculty of Medicine, University of Opole, 48 Oleska Street, 45052 Opole, Poland; 2 Department of Forensic Medicine, Faculty of Medicine, Wroclaw Medical University, 4 J. Mikulicza-Radeckiego Street, 50345 Wroclaw, Poland; 3 Department of Social Sciences and Infectious Diseases, Faculty of Medicine, Wroclaw University of Science and Technology, 27 Wybrzeze Wyspianskiego Street, 50370 Wroclaw, Poland; 4 Institute of Toxicology Research, 55/61/306 M. Curie-Sklodowskiej Street, 50369 Wroclaw, Poland

**Keywords:** homemade methcathinone, pseudoephedrine, ephedrine, intravenous administration, metabolites, isomers, UHPLC-QqQ-MS/MS, health risk

## Abstract

Intravenous use of methcathinone synthesized at home from over-the-counter medications containing pseudoephedrine or ephedrine poses significant health risks, including neurotoxicity, severe infections, and, in some cases, fatal outcomes. This study explores the public health implications of this hazardous practice. Post-mortem femoral blood and vitreous humor samples were analyzed using UHPLC-QqQ-MS/MS. The method enabled differentiation of ephedrine (a metabolite of methcathinone in this context) from pseudoephedrine (a precursor), along with the identification of relevant metabolites. A literature review was also conducted to contextualize associated health risks. The validated method accurately quantified methcathinone, pseudoephedrine, ephedrine, and identified their metabolites. The simultaneous detection of the final product and unreacted precursor supported the hypothesis of chronic intravenous use of homemade methcathinone. Literature data emphasized the risks of manganese-induced encephalopathy, injection-related infections, and the harmful effects of intravenously administered tablet excipients. These issues disproportionately affect marginalized and high-risk populations. This case highlights the diagnostic value of the method and its importance for monitoring the health impacts of illicit stimulant use. Effective responses should include public education, harm reduction strategies, surveillance of emerging drug trends, and, above all, the application of advanced analytical methods capable of comprehensive evaluation in such cases.

## 1. Introduction

Pseudoephedrine, (*S*,*S*)-2-methylamino-1-phenylpropan-1-ol, is a synthetic stereoisomer of ephedrine. It is available over-the-counter (OTC) either as the sole active substance or in combination with other agents such as painkillers or antihistamines. The maximum daily recommended dose of pseudoephedrine is 240 mg. Approximately 88% of pseudoephedrine is excreted unchanged in urine, while a minor fraction undergoes hepatic metabolism to norpseudoephedrine [1].

Pseudoephedrine, a vasoconstrictor, is used to treat rhinitis, sinus obstruction, and otitis media. Its central nervous system (CNS) effects include mood enhancement, motivation, and energy, though milder than ephedrine. Adverse effects range from anxiety and insomnia to tachycardia and arrhythmias, with potential for psychosis [1]. In 2024, the EMA’s PRAC (the Pharmacovigilance Risk Assessment Committee of European Medicines Agency) highlighted risks of pseudoephedrine-related reversible encephalopathy syndrome (PRES) and reversible cerebral vasoconstriction syndrome (RCVS)—rare but severe cerebrovascular conditions [2]. Pseudoephedrine and ephedrine can be taken as substitutes for amphetamine-like substances, and chronic stimulant abuse exacerbates systemic damage, including exerting cardiotoxic effects, increasing susceptibility to serious side effects, including RCVS and PRES [3,4].

A greater threat arises from illicit methamphetamine and methcathinone (ephedrone) synthesis from pseudoephedrine. Zuba [5] demonstrated methcathinone production from pseudoephedrine/ephedrine with potassium permanganate (KMnO_4_) and spirit vinegar in household conditions. Prolonged intravenous use of methcathinone from such reactions poses a risk of manganese poisoning, leading to manganese-methcathinone encephalopathy (MME).

The non-therapeutic use of pseudoephedrine is facilitated by its ubiquity, easy availability and relatively low cost. This hold true even within the context of individual users who produce methamphetamine/methcathinone strictly for personal use.

Synthetic cathinones, including methcathinone, exhibit routes of administration encompassing intranasal insufflation and oral ingestion. However, as of 2015, The European Monitoring Centre for Drugs and Drug Addiction (EMCDDA, currently EUDA—European Union Drugs Agency) has underscored the phenomenon of intravenous use among certain drug users. Reports indicate that most injecting cathinones previously injected heroin or amphetamines, though some long-abstinent opioid users and new injectors also initiate cathinone use. Intravenous administration is associated with complications, including injection-site burning and infectious disease transmission [6].

Considering the above, it appears necessary to not only control the market of illegal sales of preparations containing pseudoephedrine/ephedrine but also to monitor the presence of these substances, their metabolites, and the substances for which they serve as precursors (e.g., methamphetamine and methcathinone) in living drug users and in post-mortem materials, in order to determine the extent of this issue. For this reason, it is essential to utilize suitably sensitive and selective methods, including those capable of differentiating various types of isomers.

The objective of this study is to present a UHPLC-QqQ-MS/MS method that enables the quantification of post-mortem concentrations of substances such as methcathinone, pseudoephedrine, and ephedrine, in the blood and vitreous humor of individual chronically administering methcathinone intravenously. Owing to the capability of the developed method to differentiate isomers, it was possible to distinguish ephedrine (a possible metabolite of methcathinone) from pseudoephedrine (a reaction substrate in the described case) and, simultaneously, to identify metabolites of both methcathinone and pseudoephedrine. Figure S1 (in Supplementary Materials) shows main metabolism pathway of methcathinone, ephedrine and pseudoephedrine. The method significantly contributed to substantiating the hypothesis that the death resulted from intravenous administration of homemade methcathinone, as evidenced by the presence of the final product (methcathinone) and the unreacted substrate (pseudoephedrine). Another aim is to demonstrate various aspects, particularly the health complications arising from the chronic administration of methcathinone via intravenous injections, derived from pseudoephedrine-containing preparations.

## 2. Results

### 2.1. Method

The developed method enabled the discrimination between ephedrine and pseudoephedrine, as well as norephedrine and norpseudoephedrine. This not only allowed for the confirmation of the hypothesis regarding the intravenous administration of homemade methcathinone prepared from a pseudoephedrine-containing pharmaceutical product, but also facilitated the quantitative determination of both ephedrine and pseudoephedrine. In the case of the routine method, which does not allow for the differentiation between ephedrine and pseudoephedrine, only a single peak representing the sum of ephedrine and pseudoephedrine would be observed. This, on the one hand, would prevent proper interpretation of the results, and on the other, would lead to false (unreal) concentrations of either ephedrine or pseudoephedrine. These findings confirm the applicability of the developed method in forensic and clinical toxicology, as well as in other fields where isomer differentiation is essential (detailed results regarding the differentiation of isomers using the developed method are presented in the Supplementary Materials Figures S2 and S3).

### 2.2. Toxicological Results

Table 1 presents the results of toxicological analyzes in the described case. According to the partner, the man has been intravenously administering heroin for years, followed by self-administration of a homemade synthesized cathinone via intravenous route. This corroborates the findings outlined in the EMCDDA report, which underscores the notable prevalence of intravenous consumption of synthetic cathinones among individuals with prior histories of intravenous heroin or amphetamine use [6].

Upon comprehensive analysis of the gathered information, autopsy findings, and toxicological test results, the forensic pathologist who performed the autopsy determined that the individual’s cause of death was poisoning with psychoactive substances.

## 3. Discussion

The abuse of psychoactive substances remains a persistent global issue. On one hand, efforts should focus on limiting the availability of illegal drugs, on the other, as illustrated by the presented case, attention should also be directed toward identifying and preventing the misuse of substances synthesized from legally available pharmaceuticals. One essential aspect of counteracting such practices is the ability to identify them and assess their prevalence. Therefore, laboratories—particularly those operating in the fields of forensic and clinical toxicology—should be equipped with appropriate analytical methods that can serve as valuable tools in the fight against homemade substances. Detailed information regarding the value of isomer differentiation has been included in the Supplementary Materials (Section: Discussion about the value of isomer differentiation).

Intravenous administration of xenobiotics, including homemade substances, is associated with numerous health risks, such as the transmission of infectious diseases, systemic dissemination of tablet excipients, and, in the case of homemade methcathinone, the potential development of MME. A detailed discussion addressing these types of public health implications in the context of substance dependence involving intravenous use is provided in the Supplementary Materials (Section: Discussion about public health implications).

In the described case, the concentration of methcathinone in the blood was 2400 ng/mL, while in the vitreous humor, it was 2509 ng/mL. The presence of ephedrine and norephedrine may result from the metabolism of methcathinone or in addition to methcathinone use, from the intake of ephedrine itself and its metabolism to norephedrine. Reported lethal concentrations of pseudoephedrine in infants and children ranged from 1.6 to 66 mg/L (µg/mL), whereas in adults, it was 19 mg/L [7]. The determined concentration of pseudoephedrine (2701 ng/mL in blood and 859.2 ng/mL in vitreous humor) alone did not cause the man’s death. Additionally, it is crucial to consider the potential post-mortem redistribution of this substance. The qualitatively detected norpseudoephedrine is a metabolite of pseudoephedrine. As previously highlighted, this is likely the result of incomplete synthesis of methcathinone from pseudoephedrine. The efficiency of this reaction was addressed by Sikk et al. [8], who reported in their study that up to 56% of pseudoephedrine did not undergo conversion to methcathinone during the experiment.

Trazodone is an antidepressant, and the presence of *m*CPP in the biological materials of the deceased likely results from its metabolism. 7-Aminoclonazepam is a metabolite of clonazepam, a benzodiazepine used, for example, in the treatment of seizures and anxiety disorders. The concentrations of these substances determined in the analyzed materials were within therapeutic ranges and did not contribute to the cause of death. However, their presence contradicts the partner’s statement that the man was not regularly taking any medications.

As aforementioned, in the described case, the concentration of methcathinone in the blood was 2400 ng/mL, while in the vitreous humor, it was 2509 ng/mL. This represents the first report on the concentration of this substance in the context of chronic homemade methcathinone injection. Although several case reports have been published addressing the issue of homemade methcathinone abuse and its associated health consequences [8,9,10,11], in none of the 34 cases was a quantitative determination of methcathinone and its metabolites performed.

To date, only two cases have been quantitatively reported in the literature [12,13,14]. Rojek et al. [12] documented the case of a 29-year-old male, where the post-mortem concentration of methcathinone in blood was 210 ng/mL. However, it is important to note that the blood also showed 1300 ng/mL of mephedrone (4-methylmethcathinone, 4-MMC) and 2.80‰ of ethyl alcohol. The post-mortem examination in this case was conducted within 24 h of death. On the other hand, Belhadj-Tahar and Sadeg [13] reported a case involving a 29-year-old woman who fell into a coma with mydriasis and rapid respirations after consuming methcathinone dissolved in alcohol. Additionally, the woman ingested bromazepam. The blood alcohol concentration (BAC) was 0.167 g/dL, the serum methcathinone concentration was 0.50 mg/L, along with 8.89 mg/L of bromazepam and 0.19 mg/L of methylephedrine. The concentration of methcathinone in urine was 17.24 mg/L, ephedrine 11.60 mg/L, and methylephedrine 11.10 mg/L. The concentrations of the aforementioned substances were determined using HPLC-UV.

To highlight the scale of drug abuse, including intravenous use, it is essential to increase testing in patients and post-mortem cases using highly specific and sensitive methods. The lack of consistent regulations regarding testing and sample storage, particularly for underage, and the absence of mandates for laboratories to use reference methods or participate in proficiency testing and obtain accreditation, complicates the monitoring of drug abuse. The authors believe that changes in these areas will increase the detection of psychoactive substances and reveal the true scale of the problem.

It is important to note that methcathinone, like many other synthetic cathinones, is unstable in biological materials, even when samples are stored at +4 °C [7,15,16,17]. In our case, toxicological analyses were conducted over 10 days after death, so the determined concentration of this substance in the blood at the time of death may have been higher.

The issue of analyzing biological samples after extended periods is well-known to the authors. Beyond exhumation studies, which pose a distinct challenge regarding the interpretation of substance stability in such materials, the authors routinely deal with samples stored in refrigerators for long period of time or with performing tests in alternative matrices when routine materials have been previously discarded. Therefore, the authors emphasize the need to support and fund initiatives focused on studying the stability of substances in biological materials under various conditions, as well as research on the feasibility of detecting substances in alternative biological matrices.

According to the deceased’s daughter, the man allegedly injected pseudoephedrine preparations dissolved in vinegar, suggesting a home reaction similar to that described by Zuba [5]. In an experiment, Zuba used Sudafed^®^ tablets (60 mg pseudoephedrine each), potassium permanganate (KMnO_4_), and 10% spirit vinegar. After an exothermic reaction, distilled water was added, and the mixture was filtered. The resulting pale to dark yellow filtrate represented the final product of the reaction (methcathinone). Gas chromatography quantitative analysis revealed that 360 mg of pseudoephedrine (6 tablets) yielded 88.2 ± 1.8 mg of methcathinone (~14.7 mg per tablet). A similar reaction with 10 Tussipect^®^ tablets (150 mg ephedrine) produced 19.1 ± 6.5 mg of methcathinone (~1.9 mg per tablet).

This is substantiated by the presence of methcathinone in man’s biological materials, suggesting the potential utilization of potassium permanganate in the process. The production of methcathinone at home through the oxidation of pseudoephedrine with KMnO_4_ not only results in the formation of the final product but also yields solution containing manganese ions (Mn^2+^), which is another thread. To mitigate the potential for abuse of homemade methcathinone, we recommend restricting access to products containing precursors such as ephedrine and pseudoephedrine.

In cases of manganese overexposure, Mn^2+^ ions enter the bloodstream and are metabolically oxidized to Mn^3+^, which binds to transferrin and crosses the blood–brain barrier, accumulating in the basal ganglia. This process enhances dopamine auto-oxidation and free radical production [18,19].

As previously mentioned, the literature describes cases of chronic manganese due to homemade methcathinone abuse [8,9,10,11].

Manganese-methcathinone encephalopathy is a parkinsonian syndrome observed, e.g., in individuals injecting methcathinone synthesized with potassium permanganate [20]. Symptoms include spathy, dystonia, bradykinesia, speech disturbances, and gait instability, spastic-hypokinetic dysarthia. Additionally, in such cases no response to levodopa may also be observed [9,10]. Considerations regarding the feasibility and importance of manganese testing in these types of cases are discussed in Supplementary Materials (Section: Discussion about public health).

The described case concerns not only the issue of illicit substance abuse and the feasibility of homemade methcathinone synthesis from commonly available prescription medicines containing pseudoephedrine, nor only the potential occurrence of MME, but also highlights additional risks associated with intravenous drug administration, including infectious diseases and the introduction of excipients and tablet fillers directly into the bloodstream. These aspects have been discussed in detail in the Supplementary Materials (Section: Risk #2–Risk #4).

## 4. Case Report

A deceased 46-year-old male was discovered by his partner in the early morning in the basement, where he resided. The man’s body was positioned in a seated posture on a chair, with his head lowered between his knees, and his arms hanging freely to the ground. A syringe was located on the floor, next to the body. The woman acknowledged that the man had a dependency on Apselan^®^, an OTC drug containing pseudoephedrine hydrochloride, which he administered intravenously.

The woman disclosed that she had been in a relationship with the deceased for over a dozen years and that he had been addicted to psychoactive substances throughout this duration. Initially, the addiction involved heroin, and subsequently, he intravenously self-administered substances produced from purchased OTC drugs: Apselan^®^ and Sudafed^®^. The woman negated her partner’s alcohol addiction, chronic illnesses, or the use of medications for therapeutic purposes. Over the course of their relationship, the man underwent multiple treatments for drug addiction and underwent psychiatric intervention approximately two years prior to his demise.

The adult daughter of the deceased asserted that her father dissolved pseudoephedrine preparations in vinegar and administered them intravenously.

On the medial aspect of the right forearm, spanning the entire length of the venous vessel, a scar is evident, accompanied by punctate scabs observed in the proximal region. Additional scars from previous self-inflicted injuries are present on the right forearm. Examination of the aorta and coronary arteries revealed mild atherosclerosis, along with mild hepatic steatosis. No other pathological findings were noted.

The toxicological analyses commissioned by the prosecutor encompassed the determination of ethyl alcohol as well as drugs and pharmaceuticals. The prosecutor’s order did not include determinations for manganese.

The authors have no knowledge as to whether histopathological examinations were commissioned and performed in the case in question.

## 5. Materials and Methods

### 5.1. Chemicals

Water (chemsolve^®^ LC–MS), acetonitrile (chemsolve^®^ LC–MS), methanol (chemsolve^®^ LC–MS), ethyl acetate (chemsolve^®^ LC–MS), were purchased from WITKO (Łódź, Poland); formic acid and ammonium formate were purchased from Chem-Lab NV (Zedelgem, Belgium); ammonium carbonate was purchased from Fluka (Buchs, Switzerland); (1R,2S)-ephedrine, (1S,2S)-pseudoephedrine, (1S,2R)-ephedrine-d_3_ were purchased from Cerilliant (Round Rock, TX, USA), norpseudoephedrine and methcathinone were purchased from Chiron (Trondheim, Norway), norephedrine was purchased from Cayman Chemical Company (Ann Arbor, MI, USA).

### 5.2. Biological Materials and Samples Procedure

**Figure 1:**
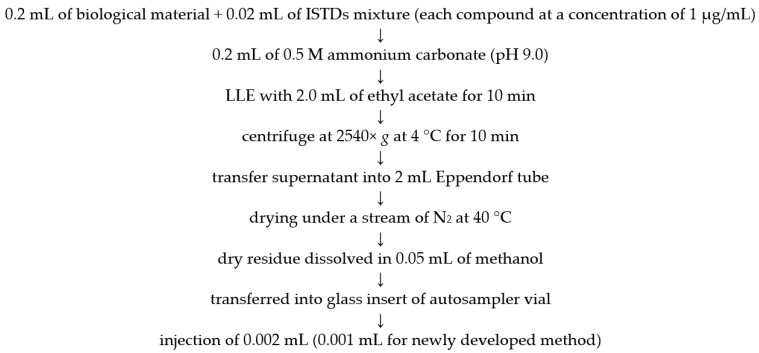
Sample preparation procedure.

Post-mortem blood and vitreous humor specimens, designated for standard toxicological analysis, were forwarded to Institute of Toxicology Research (Wroclaw, Poland). The samples underwent preparation using our routine established liquid–liquid extraction (LLE) method, incorporating the addition of pertinent internal standards (ISTDs), each maintained at a concentration of 1 µg/mL (for details please see Figure 1). The identified substances along with the appropriate internal standards are detailed in Table S1 in Supplementary Materials. Considering that concentrations of certain substances in both biological matrices surpassed the upper limit of quantification (ULOQ), a secondary preparation involved a 20-fold dilution. All samples underwent duplicate analyses. All method parameters met validation criteria, in accordance with the Scientific Working Group for Forensic Toxicology (SWGTOX) standard practices for method validation in forensic toxicology [21].

### 5.3. Chromatographic and Spectrometric Conditions

The prepared samples were analyzed using previously developed and validated UHPLC-QqQ-MS/MS methods in positive ionization MRM (multiple reaction monitoring) mode, e.g., [22,23,24]. Detailed information on chromatographic and spectrometric conditions can be found in Tables S2 and S3 in the Supplementary Materials.

To distinguish between ephedrine and pseudoephedrine, as well as norephedrine and norpseudoephedrine, an appropriate method was developed. The separation was executed using a Kinetex^®^ Biphenyl 100 Å, 2.6 μm 2.1 × 50 mm column (Phenomenex, Torrance, CA, USA), with the column oven set at 40 °C. The mobile phase consisted of a mixture of 10 mM ammonium formate in water with 0.1% formic acid (A) and mixture of 10 mM ammonium formate in methanol with 0.1% formic acid (B). The gradient elution was maintained at a constant flow rate of 0.25 mL/min. The gradient protocol was as follows: 0 min, 5% B; 7.50 min, 5% B; 8.50 min, 95% B; 8.51 min, 5% B; and these conditions were sustained for 3.5 min (total run time: 11 min). The MS parameters were as follows: nebulizing gas flow: 3 L/min, heating gas flow: 10 L/min, drying gas flow: 10 L/min, interface temperature: 300 °C, desolvation line (DL) temperature: 250 °C, heat block temperature: 400 °C. Retention time of each isomer and IS can be found in Table 2.

## 6. Conclusions

The findings of this study underscore the need for intensified monitoring of both the illegal distribution of pseudoephedrine- and ephedrine-containing products and their use in the synthesis of illicit stimulants such as methcathinone. The validated UHPLC-QqQ-MS/MS method proved to be a highly effective analytical tool for the reliable differentiation of structurally similar isomers and their metabolites. This capability was critical in reconstructing the sequence of events leading to the death in the presented case and in confirming chronic intravenous use of homemade methcathinone.

The analytical differentiation between pseudoephedrine (a precursor) and ephedrine (a possible metabolite) enabled a more accurate interpretation of toxicological findings, which is particularly important in the context of judicial proceedings and public health interventions. Moreover, the observed post-mortem concentrations provide valuable reference data for future forensic investigations involving similar substances.

This work also highlights the severe health consequences associated with chronic intravenous use of methcathinone derived from pharmaceutical preparations. The integration of analytical precision with clinical and toxicological context strengthens the argument for multidisciplinary approaches to this issue.

In conclusion, combating the public health threats posed by illicit stimulant use requires not only legal control and harm reduction strategies but also the implementation of advanced, highly selective analytical methods. These tools are essential for detecting and differentiating critical substances, understanding patterns of use, and ultimately supporting both forensic and preventive efforts.

## Figures and Tables

**Table 1:** Toxicological results in the described case.

Substance	Concentration in Blood [ng/mL]	Concentration in Vitreous Humor [ng/mL]
**Methcathinone**	2400	2509
**Pseudoephedrine**	2701	859
**Ephedrine**	300.6	98.1
**Norpseudoephedrine**	qualitative only	qualitative only
**Norephedrine**	qualitative only	qualitative only
**7-Aminoclonazepam**	37.1	4.0
**Trazodone**	906.0	279.6
***m*CPP**	32.1	2.8
**Ethyl alcohol**	0.21 [‰]	0.00 [‰]

**Table 2:** Retention time of studied isomers and IS used in the newly developed method.

Compounds	Retention Time (min)
Norephedrine	2.27
Norpseudoephedrine	2.75
Ephedrine-*d*_3_	4.45
Ephedrine	4.53
Pseudoephedrine	5.93
Methcathinone	6.56

## Data Availability

The data that supports the findings in this study are available from the corresponding authors upon reasonable request.

## Supplementary Materials

The following supporting information can be downloaded at https://www.mdpi.com/article/10.3390/ijms262411974/s1. References [9,11,19,20,25,26,27,28,29,30,31,32,33,34,35,36,37,38,39,40,41,42,43,44,45,46,47,48,49,50,51] are cited in the Supplementary Materials.

## Abbreviations

The following abbreviations are used in this manuscript:

4-MMC	4-MMC
BAC	blood alcohol concentration
CNS	central nervous system
DL	desolvation line
EMA	European Medicines Agency
EMCDDA	European Monitoring Centre for Drugs and Drug Addiction
EUDA	European Union Drugs Agency
HPLC-UV	high-performance liquid chromatography with ultraviolet detector
ISTDs	internal standards
LLE	liquid–liquid extraction
*m*CPP	*meta*-chlorophenylpiperazine, 1-(3-chlorophenyl)piperazine
MME	manganese-methcathinone encephalopathy
MRM	multiple reaction monitoring
OTC	over-the-counter
PRAC	Pharmacovigilance Risk Assessment Committee
PRES	pseudoephedrine-related reversible encephalopathy syndrome
RCVS	reversible cerebral vasoconstriction syndrome
SWGTOX	the Scientific Working Group for Forensic Toxicology
UHPLC-QqQ-MS/MS	ultra-high-performance liquid chromatography coupled with triple quadrupole mass spectrometry
ULOQ	upper limit of quantification
