# Supplementary material for: A Fatal Case Involving Chronic Intravenous Use of Homemade Methcathinone Derived from Pseudoephedrine Tablets: Post-Mortem Concentrations, Health Risk, and Medicolegal Aspect
Source: Int J Mol Sci. 2025 Dec 12;26(24):11974. doi: 10.3390/ijms262411974 (PMC12732496; doi:10.3390/ijms262411974)
Supplement: Supplementary file 1 [file ijms-26-11974-s001.zip › ijms-4021722-supplementary.pdf]

Supplementary Materials

A Fatal Case Involving Chronic Intravenous Use of Homemade Methcathinone Derived from Pseudoephedrine Tablets: Post-Mortem Concentrations, Health Risk, and Medicolegal Aspect

Karolina Nowak [1,*], Paweł Szpot [2] and Marcin Zawadzki [3,4]

[1] Department of Pharmacology, Faculty of Medicine, University of Opole, 48 Oleska Street, 45052 Opole, Poland
[2] Department of Forensic Medicine, Faculty of Medicine, Wroclaw Medical University, 4 J. Mikulicza-Radeckiego Street, 50345 Wroclaw, Poland
[3] Department of Social Sciences and Infectious Diseases, Faculty of Medicine, Wroclaw University of Science and Technology, 27 Wybrzeze Wyspianskiego Street, 50370 Wroclaw, Poland
[4] Institute of Toxicology Research, 55/61/306 M. Curie-Sklodowskiej Street, 50369 Wroclaw, Poland
* Correspondence: karolina.nowak@uni.opole.pl

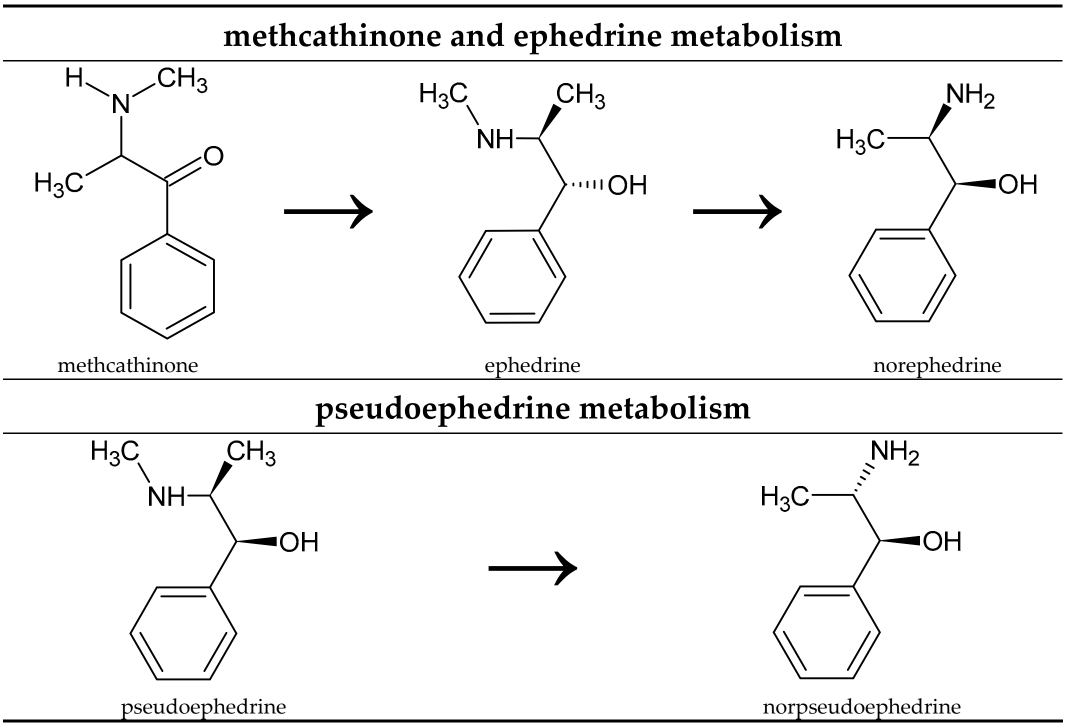

Figure S1. Methabolism pathway of methcathinone, ephedrine and pseudoephedrine.

Table S1. Quantified substances and internal standards used; *m*CPP- *meta*-chlorophenylpiperazine (1-(3-chlorophenyl)piperazine).

Substance	Internal standard
**Methcathinone**	Mephedrone-*d*3
**Pseudoephedrine**	Ephedrine-*d*3
**Ephedrine**	Ephedrine-*d*3
**7-Aminoclonazepam**	7-Aminoclonazepam-*d*4
**Trazodone**	Vilazodone-*d*4
***m*CPP**	Trifluoromethylphenylpiperazine-*d*4

Table S2. MRM condition used in routine analyses of detected compounds. * with reference to the retention time in the routine method.

Compounds	Precursor ion (*m*/*z*)	Product ion (*m*/*z*)	Q1 Pre-bias (V)	Collision energy (V)	Q3 Pre-bias (V)	Retention time* (min)
**Methcathinone**	164.10	131.10*<br>146.20	-16<br>-16	-21<br>-17	-21<br>-23	2.88
**Mephedrone-*d*$_3$**	181.00	148.20*<br>163.20	-27<br>-23	-23<br>-16	-24<br>-16	3.73
**Pseudoephedrine/Ephedrine**	166.10	148.2*<br>91.0<br>115.05	-16<br>-16<br>-16	-16<br>-33<br>-29	-25<br>-15<br>-18	2.91
**Ephedrine-*d*$_3$**	169.00	151.30*<br>115.15	-20<br>-20	-15<br>-26	-28<br>-21	2.88
**Norpseudoephedrine/Norephedrine**	152.00	134.25<br>91.15<br>115.25	-17<br>-10<br>-13	-13<br>-31<br>-25	-26<br>-16<br>-19	2.63
**7-Aminoclonazepam**	286.00	121.10<br>222.05<br>250.15*	-13<br>-13<br>-13	-30<br>-25<br>-20	-20<br>-20<br>-23	4.45
**7-Aminoclonazepam-*d*$_4$**	290.00	121.15<br>226.20<br>254.20*	-13<br>-13<br>-27	-32<br>-26<br>-22	-11<br>-22<br>-26	4.42
**Trazodone**	372.00	176.00*<br>148.00<br>78.00	-20<br>-20<br>-20	-25<br>-35<br>-50	-20<br>-20<br>-20	5.19
**Vilazodone-*d*$_4$**	446.00	155.20*<br>197.25	-20<br>-29	-46<br>-30	-14<br>-20	5.33
***m*CPP**	197.10	154.10*<br>119.15	-19<br>-10	-20<br>-24	-14<br>-19	4.40
**Trifluoromethylphenylpiperazine-*d*$_4$**	235.00	190.20*<br>217.30	-11<br>-23	-24<br>-11	-11<br>-22	4.97

Table S3. Chromatographic and spectrometric conditions of routine analyses of detected compounds.

**UHPLC system**	**Nexera X2, Shimadzu, Kyoto, Japan**	
**Column**	Kinetex XB-C18; 2.1 x 150 mm; 2.6 µm (Phenomenex, Torrance, CA, USA)	
**Column temperature**	40 °C	
**Mobile phase (A)**	10 mM ammonium formate and 0.1% formic acid in water	
**Mobile phase (B)**	0.1% formic acid in acetonitrile	
**Flow rate**	0.4 mL/min	
**Gradient**	0.0 min	5% B
	12.0 min	98% B
	14.0 min	98% B
	15.0 min	5% B
	20.0 min	5% B
**Total run time**	20.0 min	
**MS system**	Triple-quadrupole mass spectrometer (LCMS-8050, Shimadzu, Kyoto, Japan)	
**Ionization source**	Electrospray (ESI), positive	
**MS mode**	multiple reaction monitoring (MRM)	
**Nebulising gas flow**	3 L/min	
**Heating gas flow**	10 L/min	
**Drying gas flow**	10 L/min	
**Interface temperature**	250 °C	
**Desolvation line (DL) temperature**	200 °C	
**Heat block temperature**	350 °C	

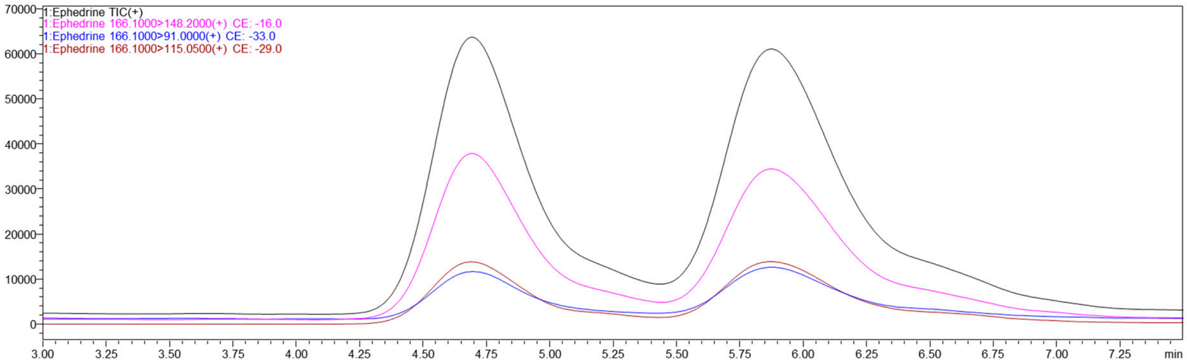

(**A**)

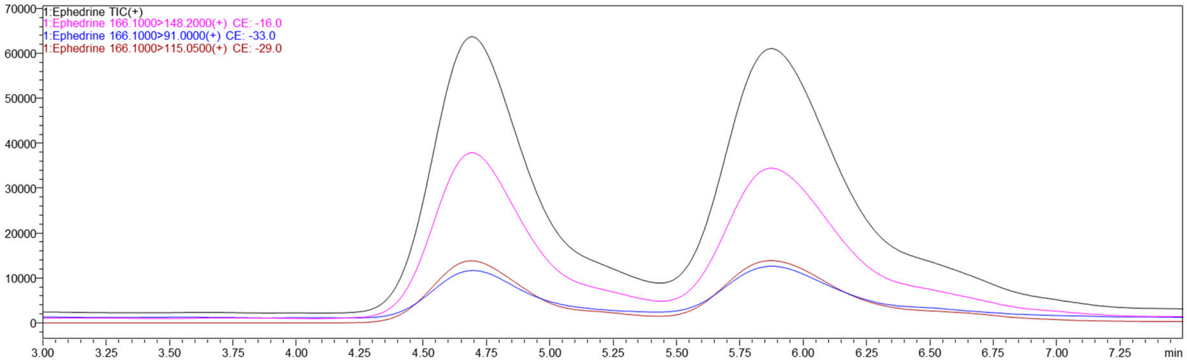

(**B**)

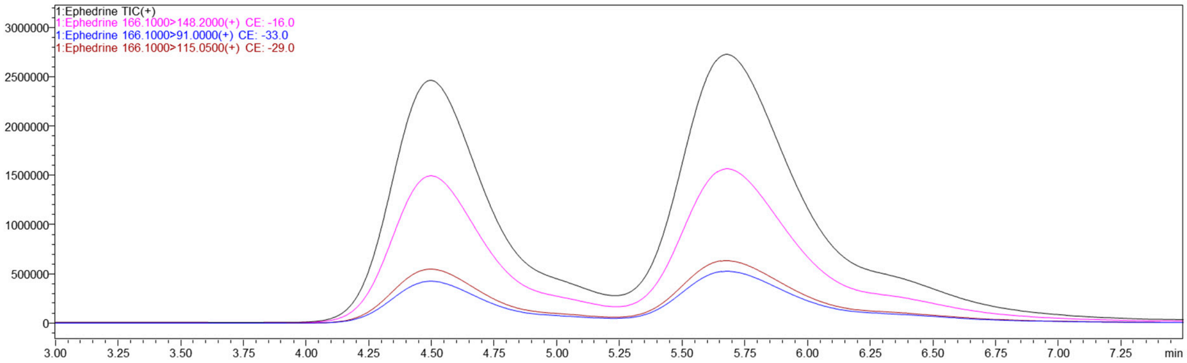

(**C**)

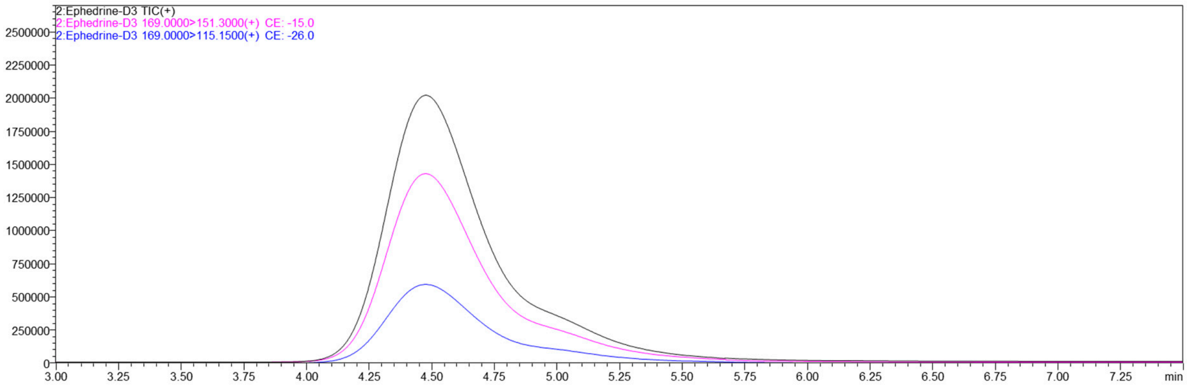

(**D**)

Figure S2. MRM transitions of mix of ephedrine (left peak) and pseudoephedrine (right peak) in concentration of 1 ng/mL (**A**), 10 ng/mL (**B**) and 100 ng/mL (**C**); MRM transitions of IS- ephedrine-d_3 in concentration of 100 ng/mL (**D**).

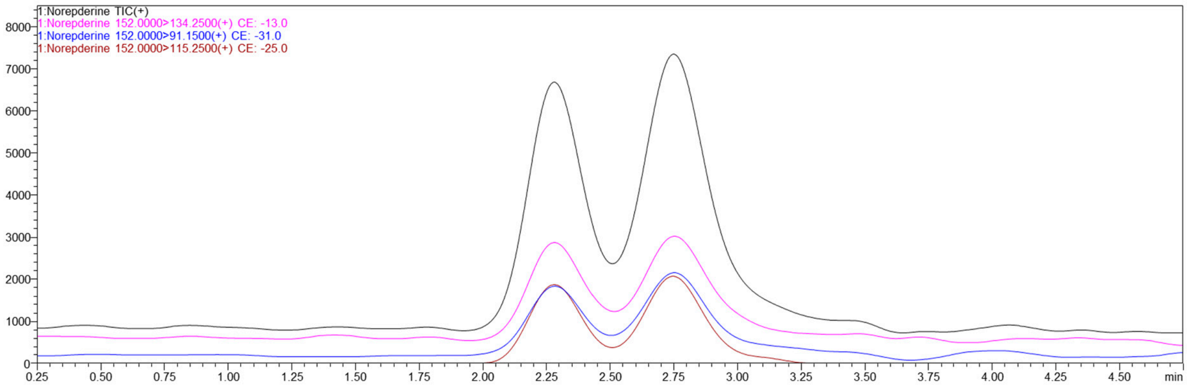

(**A**)

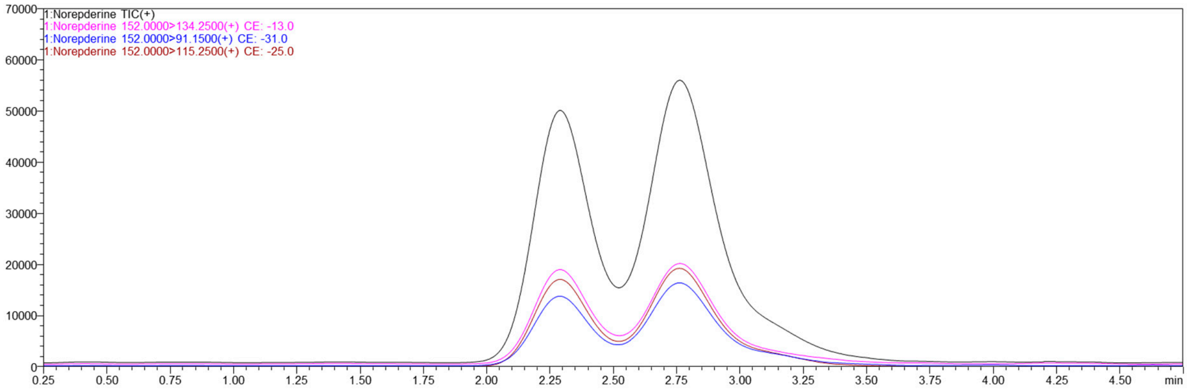

(**B**)

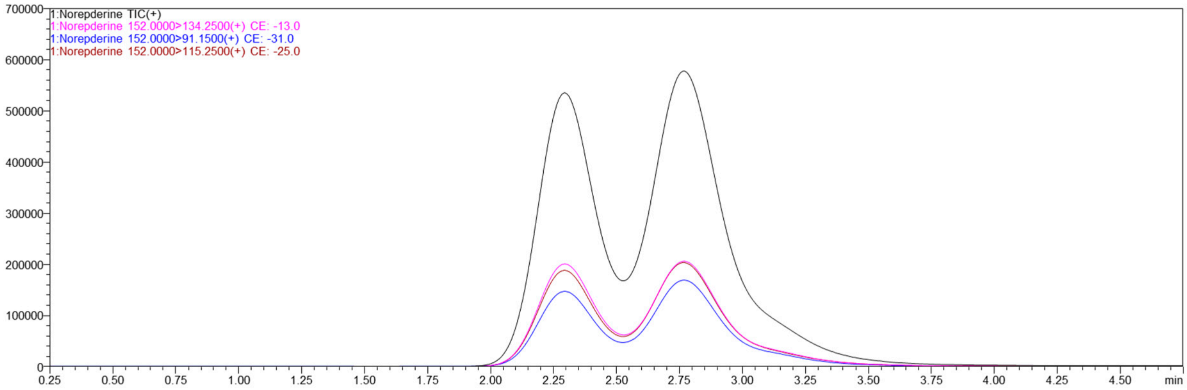

(**C**)

Figure S3. MRM transitions of mix of norephedrine (left peak) and norpseudoephedrine (right peak) in concentration of 1 ng/mL (**A**), 10 ng/mL (**B**) and 100 ng/mL (**C**).

Discussion about public health implications

Risk #1: The dangers resulting from manganese overexposure- part II, determination of manganese

In addition to clinical symptoms and a history of substance abuse, an elevated manganese concentration may support the diagnosis of MME [1–3]. Koksal et al. [3] conducted a study involving 7 patients (age range: 19-31 years) exhibiting chronic manganese toxicity resulting from prolonged intravenous use of a 'Russian Cocktail' solution composed of ephedrine, acetylsalicylic acid, and potassium permanganate. The duration of manganese exposure varied from 9 to 106 months, and the time lapse between exposure and symptom onset ranged from 7 to 35 months. The withdrawal period from manganese in three cases was 1 month, while in two other cases, it was 6 and 18 months. Two patients remained exposed during the study. Manganese serum concentrations ranged from 12 to 860 nmol (mean 456) [units as per the original manuscript]. A serum concentration of 12 nmol [units as per the original manuscript] of Mn was recorded in a patient with a manganese withdrawal period of 28 months. Observations in the patients revealed numerous symptoms, including impaired speech followed by gait disturbance and bradykinesia. Additionally, choreic movements, ataxia manifesting as backward falls, and dystonia were also observed.

In the case under discussion manganese was not analyzed postmortem An additional challenge in interpreting potential Mn results in blood would be that the majority of Mn is bound to erythrocytes, hence postmortem processes could lead to alterations in the concentration of this element. Hair analysis could offer insights into chronic exposure [4]. However, in the present case, hair samples were not collected, as in Poland, this is not a routine material obtained during autopsies, which also limits the possibilities for subsequent toxicological analyses.

Furthermore, Sikk et al. [5] point out the large variability in mean concentration of hair manganese. The authors stated that at the individual level, Mn concentrations are not reliable biomarkers of exposure due to fast elimination from plasma and urine and significant variability in hair.

The multitude of disorders caused by manganese overexposure, along with the associated increase in hospitalizations and healthcare costs for patients MME, presents a significant rationale for taking action. This includes not only restricting access to products containing ephedrine/pseudoephedrine, but also other substances involved in the chemical reaction, such as potassium permanganate. In our view, the purchase of chemical reagents should be permitted exclusively to adults, accompanied by a clear declaration regarding the intended purpose of such a purchase. Particular attention should be given to online sales platforms, where both buyers and sellers can be private individuals.

Risk #2: Infectious diseases

Intravenous use of pseudoephedrine/ephedrine-containing preparations is often accompanied by infectious comorbidities. Ennok et al. [6] reported HIV and HCV co-infection in 6 of 14 users, with the remaining eight positive for HCV alone. In the cases described by Stepens et al. [7], among 10 active methcathinone users examined, 4 individuals were AIDS positive and 5 were HIV positive. Among 13 former users, 5 were AIDS positive and 6 were HIV positive. In turn, the 36-year-old man described by de Bie et al. [8], who presented with manganese-induced parkinsonism associated with the use of homemade methcathinone, was HCV positive.

In addition to HIV, HCV, and HBV, intravenous drug users (IDUs) are at risk of acute infections due to immune suppression. These include skin and soft-tissue infections ranging from cellulitis and abscesses to necrotizing fasciitis and sepsis, commonly caused by *Staphylococcus aureus* and Group A streptococci [9]. Hengge et al. [10] described a 31-year-old HCV-positive IDU presenting with systemic inflammation, rhabdomyolysis, and tissue necrosis without fever. Severe skin damage may occur even after a single use of certain substances, such as desomorphine ('krokodil') [11].

IDUs also face an elevated risk of community-acquired pneumonia, pulmonary tuberculosis, infective endocarditis, deep vein thrombosis, osteomyelitis, septic arthritis, and even conditions such as wound botulism and anthrax

[5,12]. Vascular complications can present as damage to the vessel wall, disruption of blood flow within the lumen leading to ischemia, or hematogenous spread of a pathogen from the injection site. The incidence of vascular complications increases when individuals who inject drugs transition from injecting superficial upper limb veins to larger and deeper vessels, such as the femoral vein in the groin or the jugular vein in the neck [12].

The syringe discovered at the scene was not submitted for toxicological analysis. Information obtained from the partner and daughter does not indicate that the deceased displayed symptoms of MME before death, nor does it suggest that he had been diagnosed with HIV or Hepatitis C during his lifetime. The post-mortem examination, however, did not reveal significant alterations in the skin.

From our perspective, to limit the spread of infectious diseases, it is essential to raise public awareness about needle and syringe exchange programs for drug users and to increase funding for institutions that run these programs.

Risk #3: Systemic dissemination of tablets excipients

In cases of fatalities among IDUs injecting previously crushed and dissolved tablets, beyond the potential introduction of microorganisms through injection, it is pertinent to consider the possibility of thrombosis or an immune reaction to the presence of foreign substances. In the context of tablets, these foreign substances may include excipients, specifically insoluble particles. Excipients play a crucial role in providing the appropriate form to the drug, enhancing its stability, influencing bioavailability, and potentially improving its appearance or taste.

During post-mortem examinations of intravenous drug users, a histological examination for foreign material in various organs may prove beneficial. While this may not alter the autopsy conclusions, it represents the sole means of demonstrating systemic dissemination of excipients [13]. In the case under consideration, however, histopathological examinations were not conducted, and during the autopsy, there were no macroscopic observations of changes in organs or vessels, nor the presence of particles of insoluble excipients.

Particles of insoluble excipients or disintegrants (e.g., talc, starch, microcrystalline cellulose, crospovidone) present in the composition of tablets may deposit in the smallest branches of the pulmonary artery, leading to pulmonary embolism. This results in the onset of breathlessness and the development of pulmonary hypertension [14], with severe cases of embolism potentially leading to fatality [15]. Anderson et al. [16] described the case of a 37-year-old man who, after seven days of intravenous injection of crushed morphine tablets, experienced sharp, severe chest pain. Despite CT findings and the patient's history suggesting pulmonary granulomatosis from excipient deposition, a cardiology consultation diagnosed the patient with acute pericarditis.

Respiratory system complications may also arise from toxicity attributed to the active substance. Gilbert et al. [17] documented the case of a 34-year-old man who died to zolpidem toxicity, concurrent with pulmonary hypertension linked to intravenous injections of crushed tablets. Moreover, the cut surfaces of both the liver and spleen revealed the presence of titanium dioxide, subsequently confirmed in further examinations using scanning electron microscopy (SEM).

Reducing health complications from injecting crushed and dissolved tablets can be achieved by educating IDUs about the disorders associated with this practice. Informative talks and offering maintenance treatment that utilize alternative methods of substance administration can be helpful.

Risk #4: Selected groups of patients requiring additional supervision from (medical) personnel

Individuals chronically engaging in the injection of crushed tablets appear to be a distinct patient group requiring additional supervision from medical personnel. Shrestha et al. [18] reported a case of a 38-year-old male who presumably died after repeatedly injecting crushed tablets through a peripherally inserted central catheter during hospitalization. In the context of substance dependency, post-mortem examination unveiled enduring alterations in the impacted vascular system, characterized by smooth muscle hyperplasia of the media leading to stenosis and, in certain

vessels, obstructive occlusion of the lumen. Remarkably, numerous crystals remained densely packed within the lumina of the vessels, exhibiting no migration into or through the vessel wall or into the adjacent alveoli.

Another group at increased risk of health complications or death due to chronic intravenous drug use or the injection of crushed tablets is prisoners. Due to limited access to illegal substances, intoxication through the injection of crushed tablets can be an alternative. Prisoners undergoing methadone maintenance treatment (MMT) deserve special attention, as highlighted by Meyer et al. [19]. In Kyrgyzstan prisons, MMT and needle/syringe programs were introduced to reduce opioid dependence and the transmission of infectious diseases such as HIV or HCV infections. An unexpected outcome of these efforts was the utilization by some prisoners of provided needles/syringes for the injection of crushed tablets containing diphenhydramine, a non-prescription antihistamine substance, to achieve a state of euphoria.

Given the need for increased care for specific groups, such as addicted patients and prisoners, there is a real necessity to expand the workforce and adopt a more individualized approach to each patient. This approach aims to prevent severe complications and tailor treatments to individual needs. Encouraging people to volunteer to spend time with and assist those struggling with addiction could be beneficial.

Discussion about the value of isomer differentiation

The developed method for the determination of structural isomers enabled the quantification of key substances relevant to the described case: methcathinone, pseudoephedrine, ephedrine, norephedrine, and norpseudoephedrine. Previously published methods have generally lacked this level of completeness, focusing instead on selected compounds only—for example, pseudoephedrine alone [20], methcathinone alone [21,22], methcathinone and ephedrine [23], ephedrine and pseudoephedrine [24], pseudoephedrine, norephedrine, norpseudoephedrine, and methcathinone (excluding ephedrine) [25], or pseudoephedrine, ephedrine, norephedrine, and norpseudoephedrine (excluding methcathinone) [26].

A method allowing for the simultaneous determination of all five compounds (alongside other xenobiotics) was previously published by Beyer et al. [27] and Sørensen et al. [28].

In our method, the flow rate was set at 0.25 mL/min, compared to 1.5 mL/min in the method by Beyer et al. [27] and 0.2 mL/min in the method by Sørensen et al. [28], while the total run times were 11, 17, and 22 minutes, respectively. Considering these parameters, our method is more eco-friendly and offers cost reduction through e.g. minimal consumption of mobile phase and electricity, while also generating less chemical waste compared to the other two methods. This represents an important aspect of green analytical chemistry (GAC) [29].

When comparing retention times, the farthest among the five compounds, in our method was observed for 6.56 min (methcathinone), whereas in the method by Beyer et al. [27], methcathinone showed a retention time of approximately 7.5 minutes, and in the method by Sørensen et al. [28], methcathinone was retained at 10.12 minutes. Despite the significantly shorter overall run time of our method, it did not compromise the resolution of the analytes.

The development of analytical methods covering not only parent compounds but also their metabolites is particularly important from the perspective of forensic toxicology. This is especially relevant when substances belonging to the same class share similar metabolic pathways, including common metabolites [30,31].

Abbreviations

The following abbreviations are used in this manuscript:

CT	computed tomography
DL	desolvation line
HBV	hepatitis B virus
HCV	hepatitis C virus
HIV	human immunodeficiency virus
IDUs	intravenous drug users
LLE	liquid-liquid extraction
*m*CPP	*meta*-chlorophenylpiperazine, 1-(3-chlorophenyl)piperazine
MME	manganese-methcathinone encephalopathy
MMT	methadone maintenance treatment
SEM	scanning electron microscopy
UHPLC	ultra-high performance liquid chromatography

References

1. Iqbal, M.; Monaghan, T.; Redmond J. Manganese toxicity with ephedrone abuse manifesting as parkinsonism: a case report. *J Med Case Rep*. **2012**, *6*, 52. https://doi.org/10.1186/1752-1947-6-52.
2. Ordak, M.; Sloniewicz, N.; Nasierowski, T.; Muszynska, E.; Bujalska-Zadrozny, M. Manganese concentration in patients with encephalopathy following ephedrone use: a narrative review and analysis of case reports. *Clin Toxicol (Phila)*. **2022**, *60*, 10-17. https://doi.org/ 10.1080/15563650.2021.1973488.
3. Koksal, A.; Baybas, S.; Sozmen, V.; Sutpideler Koksal, N.; Altunkaynak, Y, et al. Chronic manganese toxicity due to substance abuse in Turkish patients. *Neurol India*. **2012**, *60*, 224-227. https://doi.org/10.4103/0028-3886.96407.
4. Habrat, B.; Silczuk, A.; Klimkiewicz, A. Manganese encephalopathy caused by homemade methcathinone (ephedrone) prevalence in Poland. *Nutrients*. **2021**, *13*, 3496. https://doi.org/ 10.3390/nu13103496.
5. Sikk, K.; Taba, P. Methcathinone “kitchen chemistry” and permanent neurological damage. *Int Rev Neurobiol*. **2015**, *120*, 257-271. https://doi.org/10.1016/bs.irn.2015.02.002.
6. Ennok, M.; Sikk, K.; Haldre, S.; Taba, P. Cognitive profile of patients with manganese-methcathinone encephalopathy. *Neurotoxicology*. **2020**, *76*, 138-143. https://doi.org/ 10.1016/j.neuro.2019.10.007.
7. Stepens, A.; Logina, I.; Liguts, V.; Aldins, P.; Eksteina, I.; Platkājis, A.; Mārtinsone, I.; Tērauds, E.; Rozentāle, B.; Donaghy, M. A Parkinsonian syndrome in methcathinone users and the role of manganese. *N Engl J Med*. **2008**, *358*, 1009-1917. https://doi.org/10.1056/NEJMoa072488.
8. de Bie, RM.; Gladstone, RM.; Strafella, AP.; Ko, JH.; Lang, AE. Manganese-induced Parkinsonism associated with methcathinone (Ephedrone) abuse. *Arch Neurol*. **2007,** *64*, 886-889. https://doi.org/10.1001/archneur.64.6.886.
9. Lavender, TW.; McCarron, B. Acute infections in intravenous users. *Clin Med (Lond*). **2013**, *13*, 511-513. https://doi.org/10.7861/clinmedicine.13-5-511.
10. Hengge, UR.; Beiderlinden, M.; Otterbach, F.; Groeben, H.; Nast-Kolb, D.; et al. 31-year-old infection drug user with massive skin necrosis and shock. *Dermatology*. **2003**, *206*, 169-171. https://doi.org/10.1159/000068458.
11. Haskin, A.; Kim, N.; Aguh, C. A new drug with a nasty bite: a case of krokodil-inducted skin necrosis in an intravenous drug user. *JAAD Case Rep*. **2016**, *2*, 174-176. https://doi.org/ 10.1016/j.jdcr.2016.02.007.
12. Delaney, FT.; Stanley, E.; Bolster F. The needle and the damage done: musculoskeletal and vascular complications associated with injected drug use. *Insights Imaging*. **2020**, *11*, 98. https://doi.org/10.1186/s13244-020-00903-5.
13. Olds, K.; Gilbert, J.; Langlois, NEI.; Byard RW. Systemic dissemination of injected foreign material. *J Forensic Sci*. **2019**, *64*, 1245-1247. https://doi.org/10.1111/1556-4029.13970.
14. Nguyen, VT.; Chan, ES.; Chou, SHS.; Godwin, JD.; Flinger, CL.; et al. Pulmonary effects of i.v. injection of crushed oral tablets: “excipient lung disease”. *AJR Am J Roentgenol*. **2014**, *203*, :W506-W515. https://doi.org/10.2214/AJR.14.12582.
15. Amass, T.; Cumplido, J.; Aswad, B.; Whittenhall, M.; Ventetuolo, C.; et al. Rapid development of pulmonary hypertension and right ventricular failure due to large vessel intravascular microcrystalline cellulosis in an intravenous drug user. *Pulm Circ*. **2020**, *10*, 2045894020907871. https://doi.org/10.1177/2045894020907871.
16. Anderson, RJ.; Corbett, B.; Ly, BT. A case of acute pericarditis following intravenous injection of crushed morphine tablets. *J Psychoactive Drugs*. **2016**, *48*, 355-358. https://doi.org/10.1080/02791072.2016.1242028.

17. Gilbert, JD.; Neubauer, K.; Byard, RW. Macroscopic identification of visceral titanium pigment in an intravenous drug user. *J Forensic Sci.* **2021**, *66*, 2024-2028. https://doi.org/ 10.1111/1556-4029.14779.
18. Shrestha, B.; McLemore, J.; Miles, M. Sudden death by acute cor pulmonare from intravenous drug abuse during an inpatient admission. Implications for unexplained in-hospital death. *Clin Pulm Med*. **2013**, *20*, 192-195. https://doi.org/10.1097/CPM.0b013e31829915b5.
19. Meyer, JP.; Culbert, GJ.; Azbel, L.; Bachireddy, C.; Kurmanalieva, A.; et al. A qualitative study of diphenhydramine injection in Kyrgyz prisons and implications for harm reduction. *Harm Reduct J*. **2020**, *17*, 86. https://doi.org/10.1186/s12954-020-00435-7.
20. Ma, M.; Feng, F.; Sheng, Y.; Cui, S.; Liu H. Development and evaluation of an efficient HPLC/MS/MS method for the simultaneous determination of pseudoephedrine and cetirizine in human plasma: application to phase-I pharmacokinetic study. *J Chromatogr B Analyt Technol Biomed Life Sci*. **2007**, *846*, 105-111. https://doi.org/10.1016/j.jchromb.2006.08.026.
21. Glicksberg, L.; Bryand, K.; Kerrigan, S. Identification and quantification of synthetic cathinones in blood and urine using liquid chromatography-quadrupole/time of flight (LC-Q/TOF) mass spectrometry. *J Chromatogr B Analyt Technol Biomed Life Sci.* **2016**, *1035*, 91-103. https://doi.org/10.1016/j.jchromb.2016.09.027.
22. Al-Saffar, Y.; Stephanson, NN.; Beck, O. Multicomponent LC-MS/MS screening method for detection of new psychoactive drugs, legal highs, in urine-experience from the Swedish population. *J Chromatogr B Analyt Technol Biomed Life Sci*. **2013**, *930*, 112-120. https://doi.org/10.1016/j.jchromb.2013.04.043.
23. Zhang, L.; Wang, ZH.; Li, H.; Liu, Y.; Zhao, M.; Jiang, Y.; Zhao WS. Simultaneous determination of 12 illicit drugs in whole blood and urine by solid phase extraction and UPLC-MS/MS. *J Chromatogr B Analyt Technol Biomed Life Sci.* **2014**, *955-956*, 10-19. https://doi.org/10.1016/j.jchromb.2014.02.007.
24. Partridge, E.; Trobbiani, S.; Stockham, P.; Scott, T.; Kostakis, C. A Validated Method for the Screening of 320 Forensically Significant Compounds in Blood by LC/QTOF, with Simultaneous Quantification of Selected Compounds. *J Anal Toxicol*. **2018**, *42*, 220-231. https://doi.org/10.1093/jat/bkx108.
25. Ammann, D.; McLaren, JM.; Gerostamoulos, D.; Beyer, J. Detection and quantification of new designer drugs in human blood: Part 2 - Designer cathinones. *J Anal Toxico*l. **2012**, *36*, 381-389. https://doi.org/10.1093/jat/bks049.
26. Song, S.; Tang, Q.; Huo, H.; Li, H.; Xing, X.; Luo J. Simultaneous quantification and pharmacokinetics of alkaloids in Herba Ephedrae-Radix Aconiti Lateralis extracts. *J Anal Toxicol*. **2015**, *39*, 58-68. https://doi.org/10.1093/jat/bku113.
27. Beyer, J.; Peters, FT.; Kraemer, T.; Maurer, HH. Detection and validated quantification of nine herbal phenalkylamines and methcathinone in human blood plasma by LC-MS/MS with electrospray ionization. *J Mass Spectrom*. **2007**, *42*, 150-60. https://doi.org/10.1002/jms.1132.
28. Sørensen, LK. Determination of cathinones and related ephedrines in forensic whole-blood samples by liquid-chromatography-electrospray tandem mass spectrometry. *J Chromatogr B Analyt Technol Biomed Life Sci*. **2011**, *879*, 727-736. https://doi.org/10.1016/j.jchromb.2011.02.010.
29. Nowak, K.; Zawadzki, M.; Jurek, T.; Postmortem determination of HbA1c and glycated albumin concentrations using the UHPLC-QqQ-MS/MS method for the purposes of medicolegal opinions. *Microchem. J.* **2020**, *155,* 104733. https://doi.org/10.1016/j.microc.2020.104733.
30. Diao, X.; Huestis, MA. New Synthetic Cannabinoids Metabolism and Strategies to Best Identify Optimal Marker Metabolites. *Front Chem*. **2019**, *7*, 109. https://doi.org/10.3389/fchem.2019.00109.
31. Lopes, RP.; Ferro, RA.; Milhazes, M.; Figueira, M.; Caldeira, MJ.; Antunes, AMM.; Gaspar H. Metabolic stability and metabolite profiling of emerging synthetic cathinones. *Front Pharmacol.* **2023**, *24*, 14:1145140. https://doi.org/10.3389/fphar.2023.1145140.
